# The Effects of Pharmacist Intervention on Emergency Department Visits in Patients 80 Years and Older: Subgroup Analyses by Number of Prescribed Drugs and Appropriate Prescribing

**DOI:** 10.1371/journal.pone.0111797

**Published:** 2014-11-03

**Authors:** Anna Alassaad, Maria Bertilsson, Ulrika Gillespie, Johan Sundström, Margareta Hammarlund-Udenaes, Håkan Melhus

**Affiliations:** 1 Department of Medical Sciences, Uppsala University, Uppsala, Sweden; 2 Uppsala University Hospital, Uppsala, Sweden; 3 Uppsala Clinical Research Center, Uppsala, Sweden; 4 Department of Pharmaceutical Sciences, Uppsala University, Uppsala, Sweden; Universidad de Valladolid, Spain

## Abstract

**Background:**

Clinical pharmacist interventions have been shown to have positive effect on occurrence of drug-related issues as well as on clinical outcomes. However, evidence about which patients benefiting most from the interventions is limited. We aimed to explore whether pharmacist intervention is equally effective in preventing emergency department (ED) visits in patients with few or many prescribed drugs and in those with different levels of inappropriate prescribing.

**Methods:**

Patient and outcome data from a randomized controlled trial exploring the clinical effects of a ward-based pharmacist intervention in patients, 80 years and older, were used. The patients were divided into subgroups according to the number of prescribed drugs (<5 or ≥5 drugs) and the level of inappropriate prescribing [using the Screening Tool Of Older People's potentially inappropriate Prescriptions (STOPP) and the Screening Tool to Alert doctors to Right Treatment (START) with a score of ≥2 (STOPP) and ≥1 (START) as cutoff points]. The effect of the intervention on the number of times the different subgroups visited the ED was analyzed.

**Results:**

The pharmacist intervention was more effective with respect to the number of subsequent ED visits in patients taking <5 drugs on admission than in those taking ≥5 drugs. The rate ratio (RR) for a subsequent ED visit was 0.22 [95% confidence interval (CI) 0.09–0.52] for <5 drugs and 0.70 (95% CI 0.47–1.04) for ≥5 drugs (p = 0.02 for the interaction). The effect of intervention did not differ between patients with high or low STOPP or START scores.

**Conclusion:**

In this exploratory study, the pharmacist intervention appeared to be more effective in preventing visits to the ED for patients who were taking fewer drugs before the intervention. Our analysis of STOPP and START scores indicated that the level of inappropriate prescribing on admission had no effect on the outcomes of intervention with respect to ED visits.

## Introduction

The increased possibility of pharmacologically treating chronic illnesses has resulted in the prescription of a high number of drugs to many individual older patients with complex co-morbidities. This increases the potential for drug-disease and drug-drug interactions [1]. A high number of prescribed drugs for any one individual has also been associated with an increased risk of inappropriate prescribing [2]–[5], medication errors [6], [7], and non-compliance with treatment [8]. The administration of ≥5 drugs at once is a common definition of polypharmacy [9]. In Sweden, all patients ≥75 years of age who are prescribed ≥5 drugs are, according to national guidelines [1], subject to an annual mandatory medication review by a general practitioner, pharmacist or nurse; similarly, in Australia, the use of ≥5 regular drugs is one of the criteria for determining a patient's eligibility for home medicines review [10]. However, more important than the actual number of prescribed drugs, is the quality of the prescribed drug treatment [11]. Inappropriate, or suboptimal, prescribing may be prescribing of more drugs than are clinically needed (overprescribing) or prescribing drugs incorrectly by prescribing the wrong drug, dose or frequency (misprescribing), but it may also be a failure to prescribe drugs that are needed (underprescribing) [9], [11]–[13]. In fact, the issue of underprescribing is often overlooked and the underuse of medications is common in patients, whether they are taking few or many medications [2]. There are a number of validated and well-studied tools for measuring the quality of prescribing. Two examples are the Screening Tool Of Older People's potentially inappropriate Prescriptions (STOPP) and the Screening Tool to Alert doctors to Right Treatment (START) [14]–[16]. These comprise sets of criteria for identification of over- or misprescribing (STOPP) and underprescribing (START).

Pharmacists, when integrated in the health care team, can help promote appropriate prescribing, and clinical pharmacist interventions have shown positive effects on overprescribing as well as mis- and underprescribing in hospitalized patients [17]–[20]. However, to our knowledge, differences in the effects of clinical pharmacist intervention on clinical outcomes between patients taking few or many drugs, or between those with a high or low level of inappropriate prescribing have not been analyzed. Our research group has previously demonstrated that the addition of a pharmacist to the health-care team at an acute internal medicine hospital ward reduces visits to the emergency department (ED) by 47%, revisits to hospital by 16%, and drug-related readmissions by 80% for patients aged ≥80 years [21]. Determination of which patients gain most benefit from pharmacist intervention, and further analysis of the elements of the intervention will allow rational designing and targeting of future interventions.

The primary aim of this study was to explore whether pharmacist intervention is equally effective in preventing subsequent visits to the ED for patients who are receiving few (<5) or many (≥5) prescribed drugs on admission. In addition, the study explored whether the effect of the intervention was consistent for patients with a high or low level of inappropriate prescribing (as measured with STOPP and START). The secondary aim was to describe the impact of the pharmacist intervention on the quality of prescribing in the <5 and ≥5 subgroups.

## Methods

### Ethics statement

Each study participant has given their written informed consent, and the study protocol was approved by the Uppsala regional ethics committee. Trial Registration: clinicaltrials.gov Identifier: NCT00661310.

### Pharmacist intervention

This study was a post-hoc subgroup analysis of patient and outcome data from a prospective, randomized, controlled study (RCT) [21] that compared patients receiving standard (non-pharmacist; control) care with those receiving enhanced, more comprehensive services where a pharmacist was part of the health-care team (intervention). The RCT has been described in detail elsewhere [21]. Briefly, 400 consecutive patients, aged 80 years or older, admitted to the acute internal medicine wards at Uppsala University Hospital were included and randomly assigned to intervention or control groups. The main elements of the intervention were medication reconciliation on admission and at discharge, a thorough medication review where drug-related problems were identified and recommendations were orally communicated to the physician in charge, patient counseling throughout the hospital stay, communication of the treatment plan to the primary care physician, and a follow-up phone call to the patients two months after discharge from hospital. The medication review was structured but none of the available tools for assessing appropriate prescribing (e.g. STOPP and START) was used prospectively. The patients were followed for 12 months after discharge from hospital and the number of revisits to hospital (ED visits and readmissions) was recorded. The number of revisits to hospital was the primary endpoint of the study. The clinical pharmacists had postgraduate clinical knowledge and training and had hospital work experience. The health-care team (for the intervention and control groups) included nurses, physicians, physiotherapists, dieticians and occupational therapists. Specialists, e.g. geriatricians, were involved as consultants when needed.

### Quality of prescribing

STOPP and START were chosen to assess the quality of the prescribing. STOPP consists of 65 criteria to identify drug-disease interactions, i.e. drugs that should be avoided in certain conditions in older people. The START criteria identify drug omissions; they contain 22 diagnoses where drug treatment should always be initiated if no contraindication is present [14]. The criteria for both STOPP and START are arranged in categories according to the relevant physiological system and each criterion can generate one point. The scores are not weighted. A high number of points, i.e. a high score, indicates a high level of inappropriate prescribing. A clinical pharmacist applied the tools to the RCT dataset retrospectively; each patient's drug therapy was assessed on the first day of the hospital stay (before the pharmacists had made any recommendations to the physician) and again on the day of discharge. The total STOPP and START score on admission and at discharge were calculated for each patient. Information required for the assessment was obtained from the patients' medication administration records [19].

### Outcome assessment

The clinical outcome variable for the primary aim in the present study was the number of visits to the ED during the 12-month follow-up period. This outcome was chosen because this was the endpoint on which the pharmacist intervention had the largest impact in the main study [21]. For the secondary aim, the changes in STOPP and START scores from admission to discharge were used to describe the effects of pharmacist intervention on the quality of prescribing for the different subgroups. In addition, the number, type and acceptance rate of the pharmacists' recommendations generated by the medication review were used to illustrate the outcomes of medication review. The types of recommendations included discontinuation of drug(s), initiation of drug therapy, changes to the drug/dosage/route, and patient counseling.

### Subgroup assignment

The patients were divided into subgroups according to the number of medications prescribed for them on admission to hospital (main analysis) and the quality of the prescriptions. The patients' regular medications, as well as the p.r.n. (as required) medications used at least once the past six months, were included. Only products with an active ingredient were considered as medications, i.e. steroid creams were included while moisturizing creams were not. In the main subgroup analysis, ≥5 drugs on admission was used as the cut-off point. This cut-off point was chosen because ≥5 drugs is a common definition for polypharmacy. Since the subgroups receiving <5 drugs and ≥5 drugs were not well balanced in size, a sensitivity analysis using the median number of drugs (eight) as the cut-off point was performed. The differential effect of the intervention between patients with a high and low level of inappropriately prescribed drugs on admission was also explored. The cut-off points used in these analyses were based on the median scores for STOPP (≥2) and START (≥1), where a higher score indicated a higher level of inappropriate prescribing.

### Statistical analysis

The baseline characteristics of the participants were summarized using frequencies for categorical variables and means with standard deviations for continuous variables. Poisson regression models, with group (intervention/control), subgroup factor, and the interaction between group and subgroup factor as independent variables, using the logarithm of time spent outside the hospital as an offset, were used for the subgroup analyses of the number of ED visits. Analyses of changes from admission to discharge in STOPP and START scores in the <5 and ≥5 drugs subgroups were performed using rank analysis of covariance [22], [23], with group (intervention/control) as a factor and score on admission as a covariate. Unpaired two-sample t-tests were used to compare the number of pharmacist recommendations between subgroups. The Chi-square test was used for categorical data (comparisons of the rate of acceptance of the pharmacist recommendations). A p-value of <0.05 was regarded as statistically significant. No adjustments for multiplicity were made, as all tests were considered to be exploratory. All analyses were carried out using SAS software (version 9.3, SAS Institute, Cary, North Carolina) and Microsoft® Excel® 2008.

## Results

Ninety patients were receiving <5 drugs and 278 were receiving ≥5 drugs on admission. The patients receiving <5 drugs on admission, irrespective of their assignment to control or intervention group, had on average fewer diagnoses and were less likely to live in nursing homes than the patients receiving a higher number of drugs. The groups did not differ substantially in any other perspective, including age (Table 1).

**Table 1:** Baseline characteristics for the patients in the <5 drugs and ≥5 drugs subgroups.

Baseline characteristics	Patients with <5 drugs (n = 90)	Patients with ≥5 drugs (n = 278)
Age, mean (SD), years	87.4 (4.08)	86.5 (4.09)
Female, No (%)	48 (53.3)	168 (60.4)
Body weight, mean (SD), kg		
Women	58.6 (12.6)	62.1 (13.5)
Men	68.8 (12.3)	72.3 (12.6)
Laboratory values, mean (SD)		
Creatinine clearance, mL/min/1.73 m^2^	43.5 (19.6)	39.3 (18)
Sodium level, mEq/L	137.7 (5.3)	137.5 (4.5)
Potassium level, mEq/L	3.9 (0.7)	4.1 (0.6)
Social support, No (%)		
Spouse or partner	25 (27.8)	79 (28.4)
Residential home	7 (7.8)	21 (21.6)
None	58 (64.4)	139 (50.0)
Number of diagnoses, mean (SD)	1.5 (1.07)	2.9 (1.48)
Medical history, No (%)		
Heart failure	13 (14.4)	128 (46.0)
Diabetes	6 (6.7)	82 (29.5)
Chronic obstructive pulmonary disease	5 (5.6)	40 (14.4)
Arrhythmia	18 (20.0)	124 (44.6)
Malignancy	18 (20.0)	43 (15.5)
Ischemic heart disease	9 (8.9)	106 (38.1)
Cerebral vascular lesion	15 (16.7)	57 (20.5)
Myocardial infarct	15 (16.7)	83 (29.9)
Hypertension	21 (23.3)	126 (45.3)
Dementia	16 (17.8)	35 (12.6)

The effect of pharmacist intervention on the number of ED visits differed significantly between the subgroups defined by the number of drugs on admission (Table 2). The patients receiving <5 drugs (main analysis) benefited more [relative rate reduction 78%; rate ratio (RR) 0.22 (95% CI 0.09–0.52)] from pharmacist intervention in this respect than those receiving ≥5 drugs (30%; RR 0.70 (95% CI 0.47–1.04)). The overall rate reduction was 47%. The sensitivity analysis confirmed our findings that the intervention was more effective for the patients receiving a lower number of drugs (RR 0.21 (95% CI 0.11–0.40) for the subgroup of patients receiving <8 drugs and 1.11 (95% CI 0.68–1.82) for those receiving ≥8 drugs).

**Table 2:** Subgroup analyses for the number of ED-visits.

	INTERVENTION GROUP				CONTROL GROUP					
Variable	Patients (n)	Person years (n)	ED Visits (n)	Rate	Patients (n)	Person years (n)	ED Visits (n)	Rate	RR (95% CI)^a^	P-value interaction^a^
**Overall effect**	182	140.9	49	0.35	186	141.0	93	0.66	0.53 (0.37–0.75)	-
**Number of drugs**										0.0175
**<5**	37	31.9	6	0.19	53	45.7	39	0.85	0.22 (0.09–0.52)	
**≥5**	145	109.0	43	0.39	133	95.2	54	0.57	0.70 (0.47–1.04)	
**Number of drugs**										<0.0001
**<8**	74	62.1	11	0.18	96	78.8	66	0.84	0.21 (0.11–0.40)	
**≥8**	108	78.8	38	0.48	90	62.2	27	0.43	1.11 (0.68–1.82)	
**STOPP score**										0.9051
**<2**	109	86.7	31	0.36	110	85.5	59	0.69	0.52 (0.34–0.80)	
**≥2**	73	54.2	18	0.33	76	53.4	34	0.64	0.54 (0.31–0.96)	
**START score**										0.2020
**<1**	133	102.4	43	0.42	126	95.9	70	0.73	0.58 (0.39–0.84)	
**≥1**	49	38.5	6	0.16	60	45.0	23	0.51	0.31 (0.12–0.75)	

a Rate ratios, 95% confidence intervals and p-values from poisson regression models with group, subgroup factor and their interaction as independent variables.

The effect of pharmacist intervention on the number of ED visits was not altered by the level of inappropriate prescribing on admission, as measured by START and STOPP (Table 2).

On admission, the intervention <5 and ≥5 drugs subgroup, had on average 0.35 and 1.70 STOPP scores respectively, and 0.38 and 0.37 START scores respectively. The control group subgroups had on average 0.55 and 1.82 STOPP scores, and 0.23 and 0.50 START scores (<5 drugs and ≥5 drugs subgroups respectively) on admission. During the hospital stay, the mean STOPP score decreased (−0.7) for the intervention group patients receiving ≥5 drugs on admission but was essentially unchanged (0.1) for the intervention group patients receiving <5 drugs. The mean START scores decreased for the intervention group (across subgroups) but were unchanged for the control group (across subgroups) during the hospital stay (Table 3).

**Table 3:** Change in STOPP and START from admission to discharge for <5- and ≥5-drugs subgroups.

	STOPP		
	Change from admission^a^		
	INTERVENTION (n = 182)	CONTROL (n = 186)	p-value^b^
<5 drugs (n = 37+53), mean (SD)	0.1 (0.6)	0.3 (0.6)	0.0089
<5 drugs (n = 37+53), median (min-max)	0 (−1, 2)	0 (−1, 2)	
≥5 drugs (n = 145+133), mean (SD)	−0.7 (1.03)	0.2 (0.8)	0.0001
≥5 drugs (n = 145+133), median (min-max)	−1 (−4, 2)	0 (−3, 3)	
All patients (n = 182+186), mean (SD)	−0.5 (1.01)	0.2 (0.7)	
All patients (n = 182+186), median (min-max)	0 (−4, 2)	0 (−3, 3)	

	START		
	Change from admission^a^		
	INTERVENTION (n = 182)	CONTROL (n = 186)	p-value^b^
<5 drugs (n = 37+53), mean (SD)	−0.4 (0.9)	0.04 (0.3)	0.0002
<5 drugs (n = 37+53), median (min-max)	0 (−4, 0)	0 (−1, 2)	
≥5 drugs (n = 145+133), mean (SD)	−0.3 (0.5)	0.04 (0.4)	<0.0001
≥5 drugs (n = 145+133), median (min-max)	0 (−2, 0)	0 (−1, 1)	
All patients (n = 182+186), mean (SD)	−0.3 (0.6)	0.04 (0.4)	
All patients (n = 182+186), median (min-max)	0 (−4, 0)	0 (−1, 2)	

SD, Standard deviation.

a Change from admission calculated as STOPP/START Score at discharge – STOPP/START Score on admission.

b p-values from rank analysis of covariance for the effect of group (Intervention or Control) on change from admission, adjusted for the score on admission.

For the subgroups receiving <5 drugs, across intervention/control groups, the mean number of drugs increased during the hospital stay (change from admission: 2.1 (SD = 2.3) for the intervention group and 1.8 (SD = 2.0) for the control group).

The STOPP category that generated most points (n = 248; 47% of total STOPP criteria) on admission was “drugs that adversely affect fallers” and in this category the criteria “prescribing of benzodiazepines” (n = 118) was the most common. A patient was in this assessment considered a faller if he/she had experienced a fall during the past year. The category of STOPP criteria for which the intervention group patients had the most relative improvement, was “musculoskeletal system”; the prescribing of non-steroidal anti-inflammatory drugs (NSAIDs) was discontinued for all intervention group patients with a history of peptic ulcer disease, moderate-to-severe hypertension, heart failure and/or chronic renal failure except for two patients (17 points on admission and 2 points at discharge). In contrast, the control group had 16 points on admission and 12 points at discharge. On admission, the START criteria category “cardiovascular diseases” generated more scores than any other category (67% of total START scores), and this was also the category in which the intervention group patients had the largest improvement (intervention group: 46 points on admission and 10 points at discharge; control group: 51 points on admission and 59 points at discharge). The individual START criterion yielding most scores was omission of an ACE inhibitor for patients with chronic heart failure (intervention group: 15 points on admission and 4 points at discharge; control group: 20 points on admission and 26 points at discharge).

The pharmacist made a total of 49 recommendations for 24 of the 37 (65%) patients in the subgroup receiving <5 drugs (no recommendations were made for the remaining 13 patients in this subgroup). The mean number of recommendations for these patients was 2.0 (SD = 1.2) and the acceptance rate (i.e. the percentage of suggested actions carried out by the physician) was 86%. For the subgroup of patients receiving ≥5 drugs (n = 145), 481 recommendations were made for 130 of the 145 (90%) patients; the mean number of suggestions was 3.2 (SD = 1.7) and the acceptance rate was 73%. The mean number of recommendations per patient was lower in the subgroup receiving fewer drugs (p = 0.001 [t = 3.29; df = 152]) and the acceptance rate appeared to be higher (p = 0.057 [χ^2^ = 3.61; df = 1]). The most common recommendations for the patients receiving <5 drugs were initiation of new drug therapy (n = 21, 43% of the total number of suggestions) and discontinuation of current drug therapy (n = 7, 14% of the total number of suggestions). For the patients receiving ≥5 drugs, the most frequent recommendations were discontinuation of current drug therapy (n = 132, 32%), initiation of new drug therapy (n = 71, 17%) and dose reduction (n = 71, 17%). The overall acceptance rate of the pharmacists' suggestions did not differ between the recommendations (p = 0.45 [χ^2^ = 0.57; df = 1], 74% for discontinuation and 70% for initiation).

## Discussion

This subgroup analysis from a randomized controlled intervention study showed that it was patients receiving a lower number of drugs who benefited most from the clinical pharmacist intervention. Since a large number of drugs often is associated with an increased risk of drug-related issues [2]–[8], the conclusion often reached, although it is not evidence-based, is that patients receiving a high number of drugs are in most need for drug-use quality improvement efforts and should be prioritized. The results from this study were therefore unexpected. Further, the analysis of the STOPP and START subgroup results found no differences in the effects of pharmacist intervention on ED visits between patients with a high and low level of inappropriate prescribing on admission to the hospital.

For the intervention <5-drugs subgroup, the number of drugs increased during the hospital stay; however, the START scores also improved for these patients. This indicates that the drugs that were added were appropriate, i.e. drugs that the patient had an indication for and that were previously omitted. Importantly, the number of drugs taken by the control <5-drugs subgroup also increased during their hospital stay, although without an improvement in START scores. This was possibly because drug therapy changes for this group during the hospital stay were mainly focused on the reason for admission, with limited attention paid to other diseases and medications, thus the overall quality of prescribing was not improved. The so-called treatment-risk paradox suggests that doctors are more unwilling to prescribe drugs to patients with polypharmacy [24]. A thorough medication review evaluates the need for new drugs and re-evaluates the need for current drugs. As it aims to avoid medications without a valid indication (even if they do not pose a risk for the patient), it decreases the incidence of unnecessary drug prescriptions that could prevent the initiation of useful drugs.

A plausible explanation to why the intervention <5-drugs subgroup did not have an improvement in STOPP, is that the number of STOPP scores in this groups was very small on admission, and a reduction therefore was difficult to obtain.

The pharmacists made more recommendations for optimizing drug therapy (both a higher frequency of recommendations, and for a larger proportion of the patients) for patients receiving ≥5 drugs on admission than for those receiving fewer drugs, but with a lower acceptance rate. This suggests that the intervention was more effective for the patients receiving fewer drugs than for those receiving more drugs in improving quality of prescribing.

The subgroup of patients who were receiving fewer drugs on admission had a lower number of co-morbidities and lower levels of social support. It has previously been demonstrated that patients living in nursing homes (i.e. has a high level of social support) generally have a higher number of drugs [25], [26] and are less involved in their drug therapy [27]. Presumably, the study patients in the <5-drugs subgroup were engaged in their drug therapy to a higher degree, and were therefore more accepting of the parts of the pharmacist intervention that aimed to improve patient knowledge and compliance to drug therapy. In addition, these patients were probably more able to communicate any perceived drug therapy issues, which would improve the quality and efficacy of the intervention. It is possible, therefore, that these patients had greater potential for benefiting from pharmacist intervention. The involvement of more primary care nurses and/or caretakers in information collection and drug counseling could thus improve the pharmacist intervention experience for patients dwelling in nursing homes, who are less involved in their own drug therapy. An alternative explanation of the results, is that patients with a high number of drugs has a greater co-morbidity burden which may limit the potential effect of a pharmacist intervention on the clinical outcome.

The pharmacists did not use STOPP and START prospectively during the medication review. Because some of the STOPP and START criteria depart from Swedish guidelines and established practice, the scores do not entirely capture the content of the medication review as performed in the study. An example is the STOPP criteria “bensodiazepines for patients with recurrent falls”. In Sweden, the bensodiazepine derivate zopiclone is the recommended drug for patients with sleeping disorders in need for medical treatment. Therefore, the pharmacists often suggested a change from a long-acting benzodiazepine (that poses a higher risk for falls and other adverse drug reactions in older people) to zopiclone; which is considered a quality improvement in prescribing, however not reflected in the STOPP scores. It should also be emphasized that drugs listed as potentially inappropriate in explicit criteria (e.g. STOPP and START) may be inappropriate for most older people but the best drug of choice for others, and drugs considered as generally appropriate can be inappropriate in certain patients and in certain situations. By mainly focusing on drugs that are listed as potentially inappropriate, the potential risk of the patient's other drugs can be underestimated [28], [29]. Explicit criteria are also often criticized for not taking the patients' co-morbidities into account [30]. The clinical pharmacists' medication reviews are based on general consensus about appropriateness of prescribing, but the recommendations are altered depending on the characteristics, health status and preferences of the individual patient. This results in a more individualized assessment. To describe the effects of the intervention in this study more accurately, a record was kept of the number, type and acceptance rate of the pharmacists' actual recommendations. Still, the strength of STOPP and START is their status as validated methods of assessing the quality of prescribing. STOPP and START also have the advantage of taking a broad view of inappropriate prescribing into account (as they estimate the level of overprescribing as well as underprescribing), which makes them superior to other available tools used to analyze inappropriate prescribing in this context. All the process assessment methods used in this study (START and STOPP assessments, and pharmacist recommendations) only reflected parts of the pharmacist intervention in the main study, i.e. the medication review. Changes in the elements of the intervention that aimed to improve patient safety and compliance and increase patients' understanding of their drug therapy were not measured and the outcomes can only be surmised.

This is a post-hoc subgroup analysis of a RCT, the trial was not powered for interaction tests and no adjustments for multiplicity were undertaken. The results should therefore be considered primarily as hypothesis generating. It should also be noted that the number of patients in each subgroup is relatively small, and therefore comparisons between the subgroups, e.g. evaluation of the impact of the pharmacist intervention, should be interpreted with caution. Yet, the subgroup effects identified in this study are clinically important; the analyses were based on a rational indication and the studied variables are valid for all hospitalized older people.

Drug-related issues, e.g. inappropriate prescribing, occur in patients irrespective of low or high numbers of prescribed drugs. Accordingly, patients receiving fewer drugs on admission to hospital should not be automatically dismissed when prioritizing who should be targeted for efforts to improve the quality of drug treatment. Our study suggests, in fact, that these patients benefit more from a clinical pharmacist intervention. Further, the results of this study do not support prioritization of patients with inappropriate prescribing (as measured with STOPP and START) for comprehensive pharmacist intervention. Future studies to test this hypothesis in patients in another age group (e.g. ≥65 years) are warranted. The elements of a ward-based pharmacist intervention that are most effective in different subgroups of patients also need to be further explored.

## Conclusions

Unexpectedly, the pharmacist intervention appeared to be more effective in preventing future visits to the ED for patients who were receiving fewer drugs on admission to hospital. Based on our analysis of STOPP and START scores, there was no difference in effect between the patients with higher and lower levels of inappropriate prescribing. The overall quality of prescribing was improved for the intervention <5 and ≥5 drugs subgroups, compared to the control group.
